# Metabolomic Analysis Using Ultra-Performance Liquid Chromatography-Quadrupole-Time of Flight Mass Spectrometry (UPLC-Q-TOF MS) Uncovers the Effects of Light Intensity and Temperature under Shading Treatments on the Metabolites in Tea

**DOI:** 10.1371/journal.pone.0112572

**Published:** 2014-11-12

**Authors:** Qunfeng Zhang, Yuanzhi Shi, Lifeng Ma, Xiaoyun Yi, Jianyun Ruan

**Affiliations:** 1 Graduate School, Chinese Academy of Agricultural Sciences, Beijing 100081, China; 2 Tea Research Institute, Chinese Academy of Agricultural Sciences, Hangzhou 310058, China; Centro de Investigación y de Estudios Avanzados, Mexico

## Abstract

To investigate the effect of light intensity and temperature on the biosynthesis and accumulation of quality-related metabolites, field grown tea plants were shaded by Black Net and Nano-insulating Film (with additional 2–4°C cooling effect) with un-shaded plants as a control. Young shoots were subjected to UPLC-Q-TOF MS followed by multivariate statistical analysis. Most flavonoid metabolites (mainly flavan-3-ols, flavonols and their glycosides) decreased significantly in the shading treatments, while the contents of chlorophyll, β-carotene, neoxanthin and free amino acids, caffeine, benzoic acid derivatives and phenylpropanoids increased. Comparison between two shading treatments indicated that the lower temperature under Nano shading decreased flavonols and their glycosides but increased accumulation of flavan-3-ols and proanthocyanidins. The comparison also showed a greater effect of temperature on galloylation of catechins than light intensity. Taken together, there might be competition for substrates between the up- and down-stream branches of the phenylpropanoid/flavonoid pathway, which was influenced by light intensity and temperature.

## Introduction

Tea (*Camellia sinensis* L.), a popular beverage with unique sensory, is an important economic source for farmers and merchants in some developing countries. The widespread consumption of tea in the world is mainly for its healthy functions including antioxidant and anti-cancer effect. The sensory quality, economic value and health functions of tea depend on the secondary metabolites in tea plant such as flavonoids (or phenolic compounds), theanine, alkaloids and others [1], [2]. All these compounds are significantly affected by environmental factors and management practices.

The biosynthesis of flavonoids in tea plants has been intensively investigated at biochemical, physiological and genetic levels. Significant progress has been made in identifying structural genes involved in the phenylpropanoid/flavonoid pathway in tea plant in recent years [3]–[7]. It is well known that synthesis and accumulation of flavonoids are strictly controlled genetically in a spatial and temporal manner and in response to a number of biotic and abiotic factors [8]. Numerous reports show high variation in levels and composition of phenolic compounds in teas from different locations, altitudes and seasons, which is often attributed to the results of changes in variety, temperature, irradiance, rainfall, nutrient or water supply [1], [9]. In addition to flavonoids, such variation has also been reported in other important quality-related compounds such as free amino acids and caffeine [10], [11].

Light intensity and temperature are two major factors that have received particular attention. Numerous studies showed that the expression of structural genes encoding biosynthesis of flavonoids and the activity of some important enzymes increased under high light intensity with a subsequent increase in concentrations of flavonoids [8], [12]. Shading of tea plants resulted in lower accumulation of phenolic compounds with improved nutritional and sensory quality [13]–[15]. However, due to the complexity of the pathway and the regulation mechanisms, sub-groups of flavonoids in tea can be differently affected by light intensity. For example, Wang et al. (2012) found that polymerization of catechins and glycosylation of flavonols might be key pathways of flavonoid metabolism in tea leaves affected by shading treatment [14].

Temperature has important effects on biosynthesis of phenolics and accumulation in a number of plant species [8], [16]. Sub-groups of flavonoids appeared to be differently affected, which might be plant species dependent. For instance, flavonol in tomato increased in response to low temperature (18–12°C) whereas grape plants grown under high temperatures (30–35°C) had significantly lower anthocyanin concentrations, while flavonol accumulation was hardly affected [17], [18]. High temperature induced a decrease in anthocyanin content in apple peel and such regulation is primarily caused by altered transcript levels of the activating regulatory complex [16]. Experimental results showing geographical and seasonal variation of phenolic compounds in teas included general climatic effects, but direct and detailed evidence relating to shading, and particularly temperature, has been very limited [10], [11].

Metabolomics analysis is a useful technology for comprehensive profiling and comparison of metabolites in biological systems, and it has been extensively used in research of plant metabolism and food science [19]. Metabolomics analysis using NMR, GC-MS and LC-MS platforms has been undertaken in tea [11], [15], [20], [21]. High resolution and high-throughput analysis techniques based on ultra-performance liquid chromatography quadrupole-time of flight mass spectrometry (UPLC-Q-TOF MS) have been receiving increasing attention and provide unique advantages for metabolomics analysis of teas [13], [21]. Metabolomics analysis has been performed to evaluate the effect of shading on tea in some recent studies [13], [15]. However, in these works the variation of temperature and its consequent effect has not been emphasized. When the amount of sun exposure was decreased by shading, the temperature is likely to be affected at the same time and it becomes a challenge to separate the confounding factors of solar radiation *per se* and temperature variations [22]. In the present work, the tea plants were shaded with two different materials providing conditions of varied light intensity and temperature. We chose to analyze fresh leaves for the metabolomics analysis to avoid changes that occur during the processing of fresh leaves to tea products [2].

## Materials and Methods

### Experimental field and shading treatments

Shading experiments was conducted in a 14-year-old tea field at a commercial plantation in Shaoxing, Zhejiang Province (SHAOXING ROYAL TEA VILLAGE CO., LTD., latitude: N 29.93, longitude: E 120.69, Runqiang Lv, Email: 877053518@qq.com). No specific permissions were required for any locations/activities. The field studies did not involve endangered or protected species. The bushes (clone Longjing 43 for green tea) were planted in double rows with inner row distance of 40 cm, outer row distance 140 cm and 33 cm space between bushes within a row. The plants were fertilized by the owner of plantation with N, P_2_O_5_ and K_2_O at levels of 600, 300 and 300 kg ha^−1^ per year, respectively, to provide adequate nutrients. During the summer tea season, tea plants were covered by Black High-density Polyethylene Tape Two-pin Net (Black Net) or Nano-insulating Film (Nano) provided by the Zhejiang Tianyuan Fabric Co., Ltd. while plants without covering served as a control. Both Black Net and Nano-insulating Film have been widely used in vegetable greenhouses and tea plantations for shading. The area of a plot (4 rows ×15 m) was 90 m^2^ and there were 4 replicates for each shading treatment and the control. The shading treatments started on July 1 when the young shoots reached a developmental stage of one bud and one leaf. After covering for 10 days until July 10, samples of young shoots of one bud with 3 leaves were taken, quickly frozen in liquid nitrogen, and stored in a −70°C ultra-refrigerator until freeze dried. Three independent sub-samples were taken from each replicated plot except in 2 plots each of the Black Net and control treatments. In these two plots only 2 independent sub-samples of the exact maturity standard could be taken at adequate quantity due to deviation in young shoot size which occurs frequently under field conditions. Consequently there were in total 11, 12 and 11 samples for Black Net, Nano-insulating Film and the control treatments, respectively. Freeze-dried samples were pulverized by a ball-miller (M301 Retsch, Germany). Five points (leaves) on the surface canopy were randomly selected from each replicate of treatments for measuring leaf temperature and light intensity. The light intensity was determined by a light meter (LiCor LI-250A) coupled to a quantum sensor (LI-190SA, Lincoln, NE). Temperature of leaf surface was measured by an infrared thermometer (CEM DT8878). The measurements were undertaken every 2 hours from 8:00 am to 18:00 pm. All measurements were repeated for 3 days and averaged data are presented.

### Determination of amino acids, chlorophylls and carotenoids

Free amino acids in young shoots samples (100 mg) were extracted by 5 mL boiling water for 5 min in 100°C water bath. Amino acid contents were measured using an automatic amino acid analyzer (Sykam S-433D, Germany) [23]. Standards were prepared from authentic reagents (Sigma-Aldrich Co., St. Louis, MO).

For measurement of chlorophylls and carotenoids, plant samples were extracted with acetone at 4°C for 16 h in the dark. Their concentrations were determined by means of HPLC (Waters 2695, Waters Corp. USA). A volume of 20 µL extract was injected into Phenomenex synergi Hydro-RP C_18_ column (250 mm×4.6 mm, 4 µm) kept at 35°C and eluted with solutions A and B with gradients running at 1 mL/min as previously reported [24]. Solution A was a mixture of acetonitrile, acetic acid, water at a volume ratio of 3∶0.5∶96.5 and B was a mixture of acetonitrile, methanol and chloroform at a volume ratio of 75∶15∶10. Solution B increased from 80% to 100% in the first 20 minutes and was then held at 100% for the next 15 minutes. Absorbance was recorded at 450 nm by a photodiode array detector (Waters 2998, Waters Corp. USA) and pigments was identified by comparing retention time and absorption spectra or authentic reagents (lutein and β-carotene, Sigma-Aldrich Co., St. Louis, MO) [25].

### Extraction of metabolites and metabolomics analysis

The metabolites in sample of young shoots were extracted with 75% methanol and 1% formic acid as described by De Vos et al. (2007). Each 0.1 g plant sample was extracted with 1 mL solvent for 10 min in an ultrasonic bath and then centrifuged at 12000 r/min for 10 min. Extracts were filtered through a 0.22 µm PTFE filter before injection for metabolomics analysis [19].

Metabolomics analysis was performed on an ultra-performance liquid chromatography (UPLC, Agilent 1290, Agilent Technologies, CA, USA) equipped with an Acquity HSS T_3_ column (1.8 µm, 100 mm ×2.1 mm, Waters Corp., Milford, MA, USA) connecting to a quadrupole-time of flight mass spectrometer (Agilent 6530 Q-TOF MS). The mobile solutions were water with 0.1% formic acid (A) and acetonitrile containing 0.1% formic acid (B) with a gradient as previously described [26]. The column was kept at 40°C and the flow rate was 0.4 mL/min. Mass spectra were acquired using electrospray ionisation over the range of m/z 100–1700. The drying gas temperature was 350°C, the cone gas flow was 50 L/h, and the desolvation gas flow was 800 L/h. The stability of the method was tested by performing 17 repeated injections of solutions prepared from authentic reagents catechins and gallic acid (Sigma-Aldrich Co., St. Louis, MO) every 2 hours. The relative standard deviation (RSD) of the retention times was below 2% and the mass error was below 1 ppm (Table S1).

### Peak identification and data processing

All LC-MS raw data files were exported without MS filtering (null values of Peak Area, Peak Height and Maximum number of peaks in MS filter) and saved as mzData (*.mzdata) using the MassHunter Workstation (B.05.00, Agilent Technologies, CA, USA). Data preprocessing was performed with the free software XCMS (standalone R Package, http://masspec.scripps.edu/xcms/xcms.php) as described (Patti, Tautenhahn, & Siuzdak, 2012; http://masspec.scripps.edu/xcms/documentation.php) [27]. The maximal tolerated m/z deviation, minimum/maximum chromatographic peak width in consecutive scans and allowable retention time deviations were set as 15 ppm, 5/20 seconds and 2 seconds, respectively. Peaks were identified on the basis of (i) actual mass (AM) and retention time (RT) and standard, (ii) AM and RT, (iii) AM and MS/MS, and (iv) AM and isotopic distribution (ID). Accurate mass and MS/MS spectral data were compared to online metabolite databases (KEGG, http://www.genome.jp/kegg/; METLIN, http://metlin.scripps.edu/; MassBank, http://www.massbank.jp) [28] and the retention time was compared to the published literature [26]. The calculation and comparison of isotopic distribution were performed using the MassHunter Workstation. The identified peaks can be further classified into Identified compounds (i and ii) and Putatively annotated compounds (iii and iv) according to the proposed minimum reporting standards for chemical analysis (Table S2) [29].

### Statistical analysis

Heat maps were generated by the statistical package of ‘ggplot2’ using the R program (http://www.r-project.org/). Univariate statistics was performed by one-way ANOVA with Tukey's post test using SPSS (version 15.0, SPSS Inc., Chicago, IL). Further statistical analyses with the 1744*34 matrix produced by XCMS were done by SIMCA-P (version 13.0, Umetrics, Umea, Sweden). Unsupervised principal component analysis (PCA) was run for obtaining a general overview of the intrinsic variance of metabolites. Based on the diversity existed in PCA and good separation of groups, the method, supervised orthogonal projection to latent structure discriminant analysis (OPLS-DA) was then used to extract maximum information from the dataset and to isolate the metabolites responsible for differences among the three treatments. Potential biomarkers for grouping were identified by analyzing the S-plot, which was declared with covariance (p) and correlation (pcorr).

## Results and Discussion

### Light intensity and air temperature above canopy in treatments

**Figure 1:**
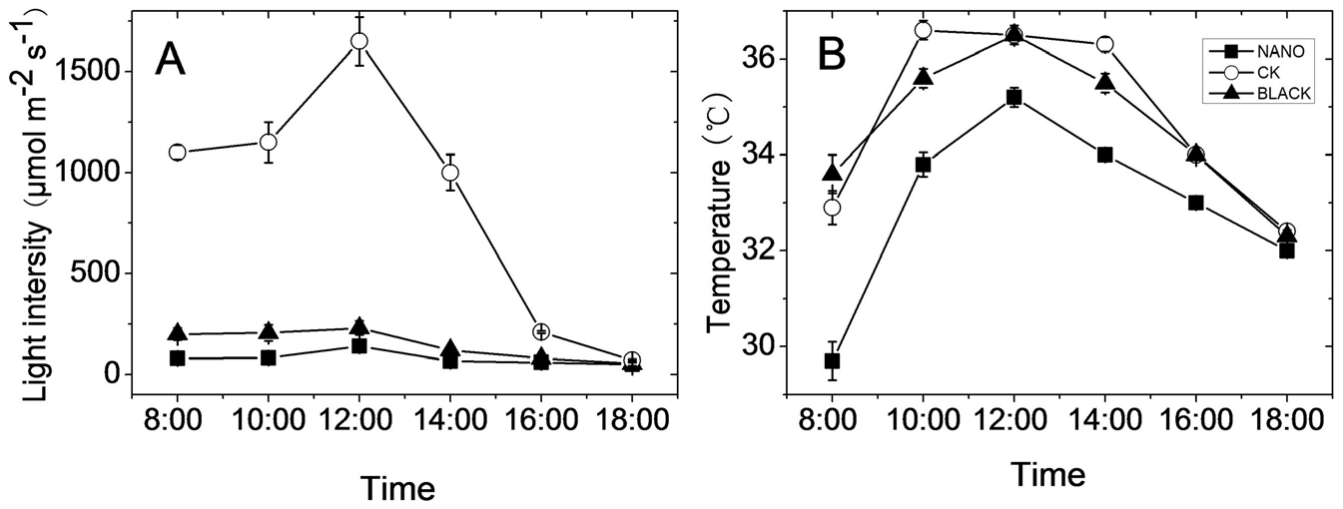
Light intensity (A) and air temperature (B) in the canopy of tea plants shaded with Black Net (Black), Nano-insulating Film (Nano) or un-shaded (CK).

Compared to the uncovered treatment (control check, CK), only 20% or even less light intensity remained on the crown of tea plants covered with Black Net and Nano-insulating Film (Fig. 1A), showing a good shading effect of both materials. The daily mean air temperature under the Nano-insulating Film was 2–4°C lower than that under Black Net while there was no significant difference (p>0.05) between the control and Black Net shading treatments (Fig. 1B). Therefore the results observed in the Black Net treatment were mainly attributed to the effect of light intensity. On the other hand, the Nano-insulating film reduced both the light intensity and temperature, allowing the effect of temperature to be judged by comparison with the Black Net treatment.

### Concentrations of free amino acids and pigments

The concentrations of free amino acids were greatly affected by the shading treatments, being significantly higher (p<0.05) in shaded plants than in the un-shaded control (Table 1). Increased contents of amino acids under shading treatments have been explained as being the result of degradation of protein induced by leaf senescence [30]. Since theanine is not incorporated into protein, its increase under shading treatments might be due to an increase in N assimilation and reduced catabolism [31].

**Table 1:** Concentrations of amino acids, chlorophylls and carotenoids in young shoots of tea plants shaded with Black Net (Black), Nano-insulating Film (Nano) or un-shaded (CK).

Compound	Treatment		
(mg g^−1^)	Un-shaded (CK)	Black shading	Nano shading
*Amino acid*			
Theanine	4.04±0.47a	9.76±3.43b	9.60±1.08b
Glutamine	0.20±0.07a	0.47±0.11b	0.35±0.11ab
Glutamate	1.01±0.03a	2.11±0.41b	2.49±0.30b
Arginine	0.30±0.07a	1.51±0.59b	1.79±0.59b
Glycine	0.11±0.01a	0.13±0.01b	0.13±0.01b
Asparate	0.78±0.03a	1.41±0.20b	1.68±0.11b
Alanine	0.18±0.05a	0.41±0.10b	0.41±0.07b
Threonine	0.09±0.01a	0.18±0.05b	0.21±0.01b
Serine	0.19±0.01a	0.49±0.14b	0.54±0.11b
γ-Aminobutyric acid	0.06±0.01a	0.09±0.02b	0.08±0.01ab
Total Amino acid	6.97±0.30a	16.56±5.00b	17.27±2.19b
*Chlorophylls and Carotenoids*			
Lutein	0.42±0.07	0.39±0.01	0.45±0.09
Neoxanthin	0.08±0.01a	0.13±0.03b	0.16±0.02b
Chlorophyll-b	0.58±0.01a	0.79±0.10ab	0.95±0.20b
Chlorophyll-a	1.99±0.29	1.65±0.15	1.94±0.23
β-Carotene	0.20±0.01a	0.29±0.01b	0.32±0.06b

Different letters following data in the same row indicate a significant difference at p<0.05.

The contents of chlorophyll-b, β-carotene and neoxanthin were significantly lower (p<0.05) in CK than in the shading treatments, whereas those of chlorophyll-a and lutein were unaffected (p>0.05 Table 1). The ratios of chlorophyll- a/b were reduced while carotenoids/chlorophyll was increased by shading treatments. The contents of pigments in the leaves under the two shading treatments were not significantly different (p>0.05), indicating that pigments in young shoots were mainly affected by light intensity and insignificantly by temperature. This response of the pigments to shade is widely known for tea and other plants [13], [14]. Previous studies showed that biosynthesis of leaf carotenoids is enhanced by light and is completely stalled under prolonged darkness [32]. However, the present work showed increased β-carotene and neoxanthin in shaded tea shoots, likely a consequence of greater carotenoid degradation exceeding the capacity of biosynthesis under high light conditions. The increased ratio between lutein and β-carotene plus neoxanthin under full sun light conditions (1.55±0.15) compared to shading treatments (0.91±0.02 for Black and 0.96±0.17 for Nano) might be a result of the greater susceptibility of the latter metabolites to chlorophyll photosensitized oxidation and radical reactions [32].

### Identification of metabolites by UPLC-Q-TOF MS

As summarized in Table S2, we identified 120 metabolites from the methanol extracts of young shoots. The main compounds were benzoic acid derivatives, flavan-3-ols, flavonols, phenylpropanoids, flavones, chalcone, flavanones and organic acids, as expected for tea leaves [15], [26]. About 90% of the metabolites were detected in negative ion detection mode with errors of 0.02–12.88 ppm. Recently, numerous studies have been performed to validate chromatographic methods to separate, detect and quantify flavonoids in teas [5], [26], [33]. Taking advantage of high resolution and efficiency, UPLC-Q-TOF/MS has been increasingly adopted to analyze metabolites in young tea shoots in a single run [34]. The results thus obtained were reliable for the further studied such as metabolic pathway analysis.

### Metabolite profiling and multivariate statistical analysis

**Figure 2:**
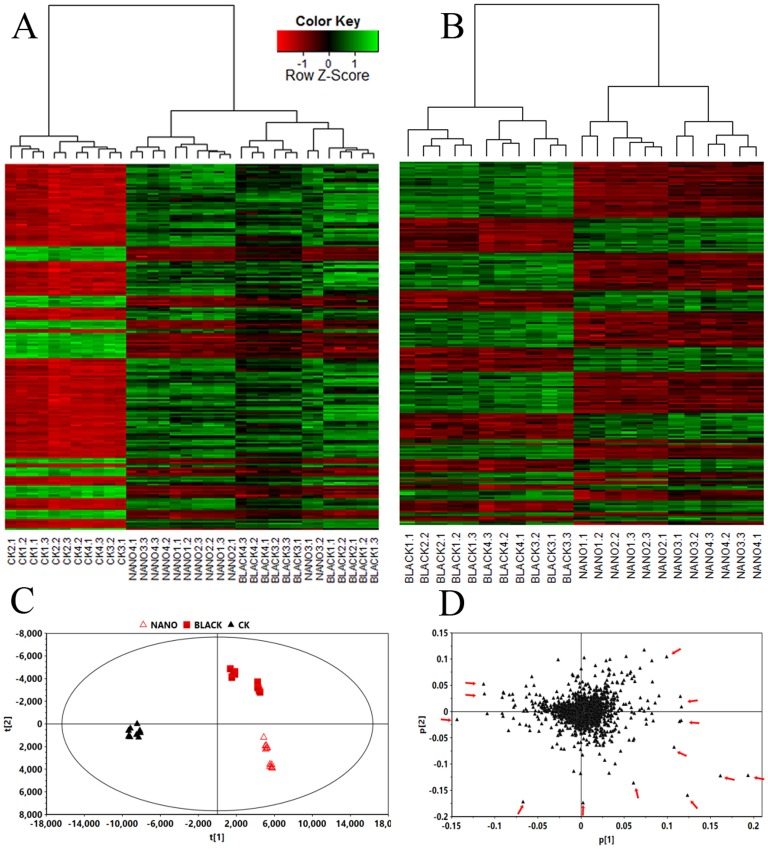
Heat maps and PCA plots of the metabolites analyzed by UPLC-Q-TOF MS in young tea shoots shaded with Black Net (Black), Nano-insulating Film (Nano) or un-shaded (CK). A: heat map (top 200 peaks ranked with p-value in XCMS output data matrix) of two shading treatments and control. B: heat map of two shaded treatments. C: unsupervised PCA score plot of shaded and unshaded treatments. D: unsupervised PCA loading plot of two shaded treatments.

1744 peaks were extracted from the chromatogram by XCMS. A heat map was generated based on the top 200 peaks and subjected to cluster analysis to provide an overview of all samples, highlighting holistic differences in the complex metabolic data (Fig. 2A, B). All samples were divided by cluster analysis accurately into independent groups, showing the difference between the un-shaded control and shading treatments, and between the two shading treatments (Nano-insulating Film and Black Net). The unsupervised PCA score plot (Fig. 2C) explained 86.3% of the total variance (R^2^) and predicted 76.3%. Samples from the control and shading treatments were clearly separated into shading and un-shading groups by PC1 (56.3%) and further into two different shading groups by PC2 (12.4%). It was observed that significantly different metabolites scattered farther away from the coordinates as indicated by the PCA loading plots from principal components 1 and 2 (Fig. 2D).

#### Metabolism affected by shading effects

**Figure 3:**
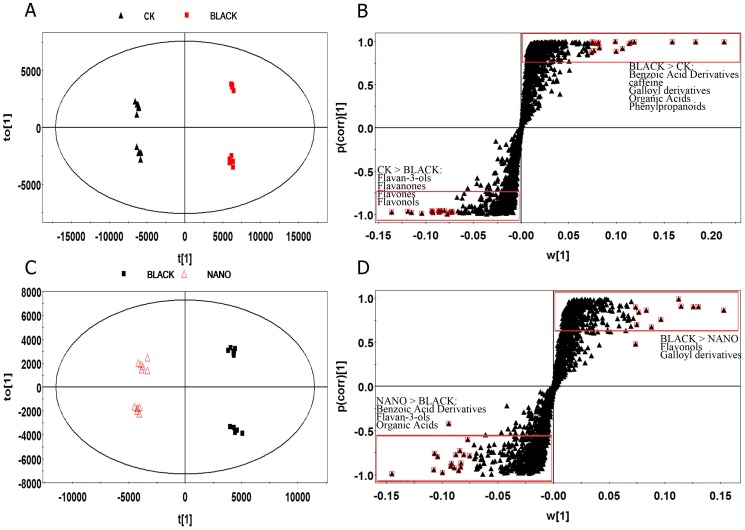
OPLS-DA score plots (A and C) and loading S-plots (B and D) of metabolites analyzed by UPLC-Q-TOF MS in young tea shoots shaded with Black Net (Black), Nano-insulating Film (Nano) or un-shaded (CK). A, B: Black Net VS un-shaded control, C, D: Nano-insulating Film VS Black Net. Red plots (the triangles in red squares) represent the 2% of compounds with the most positive or negative VIP values and P<0.001 (VIP is Variable Importance in the Projection).

**Figure 4:**
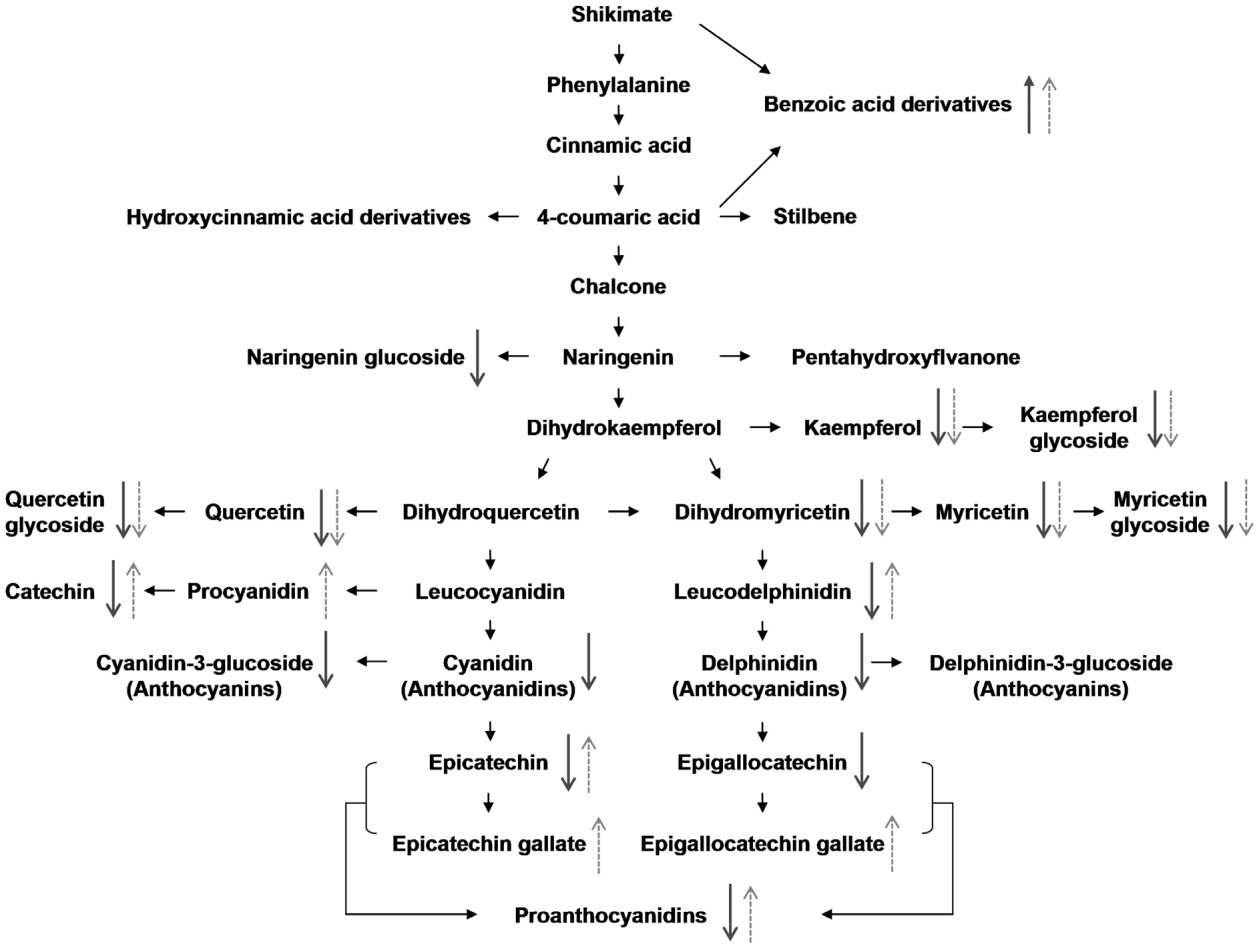
Schematic presentation of the phenylpropanoid/flavonoid pathway as affected by light intensity and temperature based on metabolomics analysis by UPLC-Q-TOF MS. Solid and dotted arrows represent the quantitative changes of metabolites with the changes of light and temperature, respectively. The up and down arrows indicate increasing and decreasing effects, respectively, of reduced light intensity by shading compared to un-shaded CK or by reduced temperature in Nano compared to Black Net.

To identify the metabolites significantly affected by shading effects, OPLS-DA modeling was performed on the profiling data sets (Fig. 3A). The model separated the un-shaded control (CK) from the samples shaded by Black Net along the discriminating t [1] for their difference in light intensity (Fig. 1). The OPLS-DA model explained more than 98% (R^2^) and predicted more than 97% (Q^2^) of the total variance. Validation carried out with CV-ANOVA (ANOVA of the cross-validated residuals) confirmed that the model had not been over-fitted (p = 6.628e-22). S-plots were constructed by presenting covariance (p) against correlation (pcorr) and the potential biomarkers for separation of shading effects were obtained by filtering with the variables important in the projection (VIP)>1 and P<0.001 in the statistical analysis. VIP is a weighted sum of squares of the PLS weight and a value >1 is generally used as a criterion to identify the important variables to the model. Although many of the significantly differing components remained unknown (Table S3), a total of 55 potential biomarkers were identified from the OPLS-DA (Fig. 3B, Table 2). Fold changes of potential markers between groups (expressed as B/CK, N/CK or N/B, Table 2) were calculated from their peak intensity to show the effect of shading. Most metabolites from the flavonoid pathway including flavan-3-ols, flavonols and glycosides, and anthocyanidins were significantly reduced (fold changes of B/CK and N/CK <1) in shading treatments (Table 1, Fig 4).

**Table 2:** Key components differentiating the two shading treatments (Black Net, B; Nano-insulating Film, N) and the un-shaded control (CK).

Compound	B/CK		N/CK		N/B	
	Fold^a^	VIP^b^	Fold	VIP	Fold	VIP
*Amino acids*						
theanine	1.96	9.28	1.85	7.85	1.16	3.87
phenylalanyl-threonine	1.89	1.96	1.74	1.65	0.92	1.23
*Organic Acids*						
nonanedioic acid	1.16	2.20	1.21	2.37	1.04	1.64
malic acid	–c	–	1.63	3.01	1.32	3.91
quinic acid	0.77	5.91	0.69	6.21	0.90	5.04
*Benzoic acid derivative & Phenylpropanoids*						
methyl gallate	1.48	3.22	2.23	4.90	1.50	6.37
m-trigallic acid	1.44	3.11	1.63	3.38	1.14	2.99
2,4-dihydroxybenzoic acid	1.21	1.08	1.29	1.28	–	–
2,5-dihydroxybenzoic acid	1.25	1.07	1.29	1.15	–	–
4-glucogallic acid	1.16	1.78	–	–	0.83	3.24
catechol	1.48	1.12	–	–	–	–
3,4-dihydroxybenzoic acid	1.18	1.15	–	–	–	–
allyl cinnamate	1.75	1.09	1.81	1.05	–	–
salicylic acid	–	–	1.27	1.47	1.12	1.81
5-O-caffeoylquinic acid	0.79	1.62	0.75	1.63	–	–
theogallin	0.96	1.06	–	–	–	–
4-caffeoylquinic acid	–	–	–	–	1.12	3.01
*Flavanones*						
naringenin-7-O-glucoside	0.52	1.99	0.51	1.82	–	–
prunin 6″-p-coumarate	0.88	1.39	–	–	1.18	2.4
*Flavones*						
7-methoxyflavone	0.59	2.80	0.63	2.41	–	–
*Flavonols & glycosides*						
quercetin 3-(3R-glucosylrutinoside)	0.74	4.53	0.67	4.63	0.90	3.36
isoquercitrin	0.51	3.38	0.43	3.28	0.84	1.94
morin	0.73	2.34	0.56	2.81	0.76	2.73
myricetin	0.52	2.02	0.47	1.93	0.89	1.3
quercetin	0.51	1.87	0.42	1.84	0.82	1.06
kaempferol 3-(4″-caffeyllaminaribioside)-7-rhamnoside	0.26	1.97	0.18	1.88	0.68	1.61
quercetin-3-Glc-Ara	0.81	1.85	0.63	2.70	0.78	2.94
kaempferol-3-O-rutinoside	0.75	1.84	0.66	1.98	0.88	1.57
dihydromyricetin	0.66	1.81	0.59	1.80	0.89	1.2
myricetin 3-sambubioside	0.77	4.08	0.78	3.66	–	–
kaempferol 3-β -d-glucopyranoside	0.57	3.51	0.55	3.27	–	–
p-Coumaroyl quinic acid	0.74	2.43	0.74	2.16	–	–
quercetin 3,4′-diglucoside	0.65	1.87	0.61	1.76	–	–
quercetin 3-xyloside-7-glucoside	0.76	1.87	0.78	1.70	–	–
quercetin-3-O-rutinoside	1.36	4.95	1.39	4.67	1.02	1.49
*Flavan-3-ols*						
epigallocatechin (EGC)	0.77	5.17	0.78	4.57	–	–
gallocatechin (GC)	0.71	4.05	0.70	3.71	–	–
catechin 7-O-alpha-L-rhamnopyranoside	0.49	1.34	0.48	1.25	–	–
catechin-4-ol 3-O-beta-D-galactopyranoside	0.43	1.91	0.46	1.68	–	–
catechin	0.71	3.16	0.81	2.27	1.14	1.33
epicatechin (EC)	0.72	3.15	0.77	2.54	1.08	1.42
leucodelphinidin	0.73	2.69	0.81	2.03	1.10	1.7
catechin 3-O-rutinoside	0.78	1.30	0.69	1.42	0.88	1.16
catechin 5,7,-di-O-gallate	0.89	1.56	–	–	1.12	2.37
catechin gallate	0.97	1.01	–	–	1.05	2.52
epigallocatechin gallate (EGCG)	–	–	1.06	2.79	1.05	3.69
gallocatechin gallate (GCG)	–	–	1.12	1.51	1.04	3.03
*Proanthocyanidin*						
epicatechin-epigallocatechin 3-O-gallate	0.74	2.12	0.77	1.78	–	–
procyanidin	–	–	1.07	1.80	1.08	3.3
cinnamtannin A1	–	–	–	–	1.15	1.27
ent-epicatechin-ent-epicatechin 3′-gallate	–	–	–	–	1.22	1.63
*Anthocyanidins*						
pelargonidin	0.19	2.90	0.13	2.70	–	–
cyanidin 3-(6″-caffeylglucoside)	0.47	3.63	0.48	3.24	–	–
cyanidin-3-O-(6″-O-malonyl-2″-O-glucuronyl) glucoside	–	–	–	–	1.25	1.02
*Alkaloids*						
caffeine	1.12	4.64	1.15	4.83	1.03	2.93

a The fold change value is based on comparing the peak intensity (content of metabolites) between different treatments (groups).

b VIP is Variable Importance in the Projection. Key components were obtained by filtering with the VIP>1 and P<0.001 in the statistical analysis.

c Represent the components with VIP<1 or P>0.001 in the statistical analysis.

Reduced biosynthesis and accumulation of flavonoids (flavonols, flavan-3-ols and anthocyanins) under low irradiance or shading conditions has been widely reported. Under shaded conditions, expression of structural genes encoding enzymes of the flavonoid pathway in tea was significantly down-regulated and the expression levels were closely correlated with concentrations of O-glycosylated flavonols and proanthocyanins [14]. Recent studies identified a number of transcription factors which activate or repress the expression of structural genes involved in anthocyanin accumulation in response to light conditions [8], [35], [36]. On the other hand, the accumulation of phenolic compounds was probably affected by the carbon or sugar status of the leaves as well [37], [38]. It has been argued that shading reduces photosynthesis, and hence the production of substrates for secondary metabolism, leading to decreasing accumulation of flavonoids. However, a few flavonoids were not significantly reduced (e.g. EGCG, GCG) or even increased (e.g. quercetin-3-O-rutinoside) by shading, reflecting the fact that genes might be differently regulated by light intensity.

In contrast to the decreasing accumulation of flavonoids, caffeine, benzoic acid derivatives (e.g. methyl gallate and m-trigallic acid) and allyl cinnamate increased in shading treatments (Table 2). Similar observations have been reported in other experiments. For example, Yang et al. (2012) found a marked increase of phenolic acids in shaded tea leaves while there were more phenylpropanoids/benzenoids and lower catechins in etiolated tea leaves. These results suggest that there might be competition for substrates between upstream and down-stream branches of the phenylpropanoid pathway in tea plants under different light intensity (Fig 4) [30], [39]. Yang et al. (2012) suggested that metabolism from phenylalanine/cinnamate to phenylpropanoids/benzenoids contributes to produce electrons for reduction- oxidation reactions in secondary metabolic pathways by recycling NADPH (NADH) and NADP^+^ (NAD^+^). Catechins biosynthesis mainly relates to the formation of oxidised derivatives of phenols by P450 enzymes.

#### Effects of temperature in addition to shading treatment

To explore the temperature effect during shading, another multivariate statistical analysis was performed on the datasets from the two different shading treatments. Again the two groups of samples were well separated by the discriminating t [1] for their difference in temperature (Fig 3C). The OPLS-DA model explained more than 96% (R^2^) and predicted more than 95% (Q^2^) of the total variance with a p-value of 3.08e-17 by CV-ANOVA. Analysis of the S-plot (Fig. 3D) showed that most flavonols and glycoside increased (fold changes N/B<1) at higher temperature under Black Net shading; while benzoic acid derivatives (excluding 4-glucogallic acid), some flavan-3-ols (excluding catechin 3-O-rutinoside) and proanthocyanidins increased (fold changes N/B>1) at lower temperature under Nano-insulating Film shading (Table 2, Fig 4). Procyanidin, EGCG, and GCG were not significantly different between the groups of Black net shading and CK but contributed significantly to distinguishing the two shading treatments, implying that metabolism of these compounds might be largely affected by temperature. On the other hand, a few flavonols and their glycosides (notably myricetin 3-sambubioside, kaempferol 3-β-d-glucopyranoside and p-Coumaroyl quinic acid), flavano-3-ols (notably EGC, GC), anthocyanidins (pelargonidin and cyanidin 3-(6″-caffeylglucoside) differed significantly between shading and un-shading treatments, but not between the two shading treatments, possibly suggesting greater sensitivity to change of light intensity than to temperature (Fig 4). It is interesting to note that the values of VIP for gallated catechins (CG, GCG, EGCG and catechin 5,7-di-O-gallate) and proanthocyanidins were much larger for comparisons between the two shading treatments than between shading and un-shading treatments (Black vs CK or Nano vs CK), showing a greater influence of temperature. By contrast, values of VIP for non-gallated catechins (C, EC, GC, and EGC) were much greater for comparisons between shading and un-shading treatments, showing a larger effect of light intensity. Therefore it appeared that galloylation of catechins was more affected by temperature than by light intensity although the mechanism has not been clearly understood [40]. The present result was in line with some other findings that biosynthesis of galloylated catechins was less significantly affected by light intensity [6], [14]. However, due to limitations in managing temperature under field conditions, the effect of temperature observed here is indirect and needs more work in the future. Some recent studies showed that expression of structural genes encoding flavonoid biosynthesis enzymes and related transcription factors in grape were regulated independently but in a synergistic way by temperature and light [36], [41]. These results highlight new clues for further study to elucidate the effects of temperature and light, alone or synergistically, on phenylpropanoid/flavonoid pathway in tea plants.

## Conclusions

In summary, the present work uncovers the effects of light intensity and temperature under shading treatments on the metabolites in tea. We found that shading reduced the accumulation of flavonoids but increased upstream metabolites, benzoic acid derivatives and free amino acids. Moreover lower temperature decreased flavonols and their glycosides but increased accumulation of flavan-3-ols and proanthocyanidins. The comparison also showed galloylation of catechins was influenced by temperature to a greater extent than by light intensity. Taken together, the present results demonstrated that there might be competition for substrates between the up- and down-stream branches of the phenylpropanoid/flavonoid pathway, which was influenced by light intensity and temperature.

## Supporting Information

Table S1 **Stability of the MS measurements.**
(DOC)

Table S2 **Metabolites identified in methanol extracts of tea leaves.**
(DOC)

Table S3 **The unidentified key components differentiating the two shading treatments (Black Net, B; Nano-insulating Film, N) and the un-shaded control (CK).**
(DOC)
