# Supplementary material for: Metabolomic Analysis Using Ultra-Performance Liquid Chromatography-Quadrupole-Time of Flight Mass Spectrometry (UPLC-Q-TOF MS) Uncovers the Effects of Light Intensity and Temperature under Shading Treatments on the Metabolites in Tea
Source: PLoS One. 2014 Nov 12;9(11):e112572. doi: 10.1371/journal.pone.0112572 (PMC4229221; doi:10.1371/journal.pone.0112572)
Supplement: Table S1 — Stability of the MS measurements. (DOC) [file pone.0112572.s001.doc]

Table S1 Stability of the MS measurements.

| Metabolite | Mass（tag） | RT (s) | STD（Rt） | Precursor (+) and (-) | Mass | Diff (ppm) |
| --- | --- | --- | --- | --- | --- | --- |
| catechin | 290.079 | 178.67 | 0.0970 | 291.0861 | 290.0788 | -0.6676 |
|  |  |  |  | 289.0719 | 290.0791 | 0.4383 |
| catechin gallate | 442.09 | 278.43 | 0.6204 | 443.0973/465.0789 | 442.0903 | 0.7305 |
|  |  |  |  | 441.0832/883.1728 | 442.0900 | 0.6283 |
| epicatechin | 290.079 | 211.00 | 0.0899 | 291.0862 | 290.0790 | -0.0241 |
|  |  |  |  | 289.0718 | 290.0790 | 0.6283 |
| epigallocatechin | 306.074 | 160.70 | 0.0966 | 307.0814 | 306.0741 | 0.4558 |
|  |  |  |  | 305.0671/611.1407 | 306.0740 | 0.3550 |
| epigallocatechin gallate | 458.0849 | 215.20 | 0.1788 | 459.0923 | 458.0852 | 0.5831 |
|  |  |  |  | 457.0777/915.1624 | 458.0850 | 0.2275 |
| gallocatechin | 306.074 | 121.51 | 0.1560 | 307.0814 | 306.0742 | 0.7129 |
|  |  |  |  | 305.0667/611.1411 | 306.0741 | 0.6283 |
