# Supplementary material for: Metabolomic Analysis Using Ultra-Performance Liquid Chromatography-Quadrupole-Time of Flight Mass Spectrometry (UPLC-Q-TOF MS) Uncovers the Effects of Light Intensity and Temperature under Shading Treatments on the Metabolites in Tea
Source: PLoS One. 2014 Nov 12;9(11):e112572. doi: 10.1371/journal.pone.0112572 (PMC4229221; doi:10.1371/journal.pone.0112572)
Supplement: Table S2 — Metabolites identified in methanol extracts of tea leaves. (DOC) [file pone.0112572.s002.doc]

Table S2 Metabolites identified in methanol extracts of tea leaves.

| Compound | RT  (min) | Exact mass | MZ | Actual mass | Error  (ppm) | Reference |
| --- | --- | --- | --- | --- | --- | --- |
| *Amino acids* |  |  |  |  |  |  |
| theanine | 0.91 | 174.1004 | 175.1073 | 174.1000 | 2.30 | MS/MS^a^ |
| L-tyrosine | 1.05 | 181.0739 | 180.0659 | 181.0730 | 4.81 | AM & Rt^b^ |
| phenylalanine | 1.78 | 165.0790 | 164.0695/166.0677 | 165.0778 | 10.33 | MS/MS |
|  |  |  |  |  |  |  |
| *Organic Acids* |  |  |  |  |  |  |
| malic acid | 0.87 | 134.0215 | 133.0151 | 134.0224 | 9.18 | MS/MS |
| shikimic acid | 3.43 | 174.0528 | 173.0450 | 174.0523 | 0.49 | AM & ID(98.51) ^c^ |
| nonanedioic acid | 6.29 | 188.1049 | 187.0976 | 188.1049 | 0.13 | MS/MS |
| quinic acid | 0.81 | 192.0634 | 191.0584 | 192.0657 | 11.98 | MS/MS |
|  |  |  |  |  |  |  |
| *Benzoic acid derivatives* |  |  |  |  |  |  |
| catechol | 3.06 | 110.0368 | 109.0295 | 110.0367 | 0.03 | AM & Rt |
| 3-hydroxybenzaldehyde | 4.17 | 122.0368 | 121.0293 | 122.0366 | 1.64 | AM & Rt |
| benzoic acid | 4.70 | 122.0368 | 123.0428 | 122.0359 | 6.75 | MS/MS |
| anthranilic acid | 4.15 | 137.0477 | 138.0553 | 137.0483 | 4.38 | AM & Rt |
| salicylic acid | 6.09 | 138.0317 | 137.0246 | 138.0318 | 0.72 | AM & Rt |
| 4-hydroxybenzoic acid | 2.68 | 138.0317 | 137.0239 | 138.0312 | 3.62 | AM & Rt |
| 2,5-dihydroxybenzoic acid | 3.16 | 154.0266 | 153.0192 | 154.0264 | 1.30 | AM & Rt |
| 3,4-dihydroxybenzoic acid | 2.01 | 154.0266 | 153.0193 | 154.0267 | 0.02 | AM & Rt |
| 2,4-dihydroxybenzoic acid | 3.48 | 154.0266 | 153.0190 | 154.0262 | 2.81 | AM & Rt |
| vanillic acid | 3.11 | 168.0423 | 167.0355 | 168.0428 | 3.24 | AM & Rt |
| gallic acid | 1.43 | 170.0215 | 169.0138 | 170.0211 | 2.35 | Standard^d^ |
| 4-aminophenylalanine | 2.01 | 180.0899 | 181.0972 | 180.0900 | 0.34 | AM & ID(98.51)^c^ |
| methyl gallate | 3.14 | 184.0372 | 183.0321 | 184.0394 | 11.95 | AM & Rt |
| allyl cinnamate | 2.54 | 188.0837 | 189.0904 | 188.0829 | 4.22 | AM & ID(93.96) |
| phenylalanyl-threonine | 2.51 | 266.1267 | 265.1195 | 266.1267 | 0.02 | AM & ID(99.18) |
| salicin | 4.79 | 286.1053 | 287.1113 | 286.1040 | 4.38 | AM & ID(91.31) |
| ellagic acid | 4.57 | 302.0063 | 300.9985 | 302.0059 | 0.40 | AM & ID(98.06) |
| digallate | 2.94 | 322.0325 | 321.0259 | 322.0334 | 0.88 | AM & ID(96.45) |
| 4-glucogallic acid | 1.53 | 332.0743 | 331.0664 | 332.0737 | 0.61 | AM & ID(96.74) |
| theogallin | 1.43 | 344.0743 | 687.142/343.0681 | 344.0744 | 0.08 | AM & ID(95.47) |
| m-trigallic acid | 3.32 | 474.0434 | 473.0412 | 474.0485 | 10.76 | AM & Rt |
| 1,3-dicaffeoylquinic acid | 5.62 | 516.1268 | 515.1212 | 516.1285 | 1.73 | AM & ID(93.42) |
|  |  |  |  |  |  |  |
| *Phenylpropanoids* |  |  |  |  |  |  |
| p-coumaric acid | 2.83 | 164.0473 | 163.0401 | 164.0472 | 0.08 | AM & Rt |
| caffeic acid | 3.35 | 180.0423 | 181.0498 | 180.0420 | 1.67 | AM & Rt |
| caftaric acid | 0.67 | 312.0481 | 351.0101 | 312.0478 | 0.97 | AM & ID(54.07) |
| fertaric acid | 3.23 | 326.2500 | 344.2800 | 326.2477 | 7.05 | AM & ID(82.56) |
| neochlorogenic acid | 2.31 | 354.0951 | 353.0871 | 354.0950 | 0.28 | AM & Rt |
| cryptochlorogenic acid | 2.94 | 354.0951 | 353.0887 | 354.0958 | 1.98 | AM & Rt |
|  |  |  |  |  |  |  |
| *Coumarins* |  |  |  |  |  |  |
| 4-hydroxycoumarin | 6.27 | 162.0317 | 161.0238 | 162.0311 | 3.70 | AM & Rt |
| esculetin | 3.38 | 178.0266 | 177.0195 | 178.0269 | 1.69 | AM & Rt |
| *Stilbenes* |  |  |  |  |  |  |
| 4-hydroxystilbene | 5.46 | 196.0888 | 197.0956 | 196.0881 | 3.47 | AM & ID(95.78) |
| cis-piceid | 6.83 | 390.1315 | 429.0962 | 390.1302 | 3.33 | AM & Rt |
| astringin | 8.58 | 406.1264 | 405.1195 | 406.1266 | 0.52 | AM & ID(98.73) |
| cis-viniferin | 6.90 | 454.1416 | 493.1026 | 454.1397 | 4.18 | AM & Rt |
|  |  |  |  |  |  |  |
| *Chalcone* |  |  |  |  |  |  |
| chalcone | 8.13 | 208.0888 | 209.0970 | 208.0892 | 1.73 | AM & ID(78.37) |
| dihydrochalcone | 8.55 | 210.1045 | 211.1127 | 210.1053 | 3.86 | AM & ID(80.95) |
| 4'-hydroxy-2'-methoxychalcone | 11.24 | 254.0943 | 253.0875 | 254.0947 | 1.41 | AM & ID(98.35) |
| 4,2',4',alpha-tetrahydroxydihydrochalcone | 4.13 | 274.0841 | 273.0769 | 274.0842 | 0.09 | AM & ID(99.83) |
| 3,4,5,2',4',6',beta-hexahydroxychalcone 2'-glucoside | 5.69 | 466.1111 | 465.1041 | 466.1114 | 0.70 | AM & ID(96.48) |
|  |  |  |  |  |  |  |
| *Flavanones* |  |  |  |  |  |  |
| naringenin | 4.71 | 272.0685 | 273.0723 | 272.0650 | 12.86 | MS/MS |
| eriodictyol | 8.24 | 288.0634 | 287.0557 | 288.0628 | 2.08 | AM & Rt |
| paratocarpin A | 3.44 | 388.1675 | 387.1591 | 388.1660 | 3.98 | AM & ID(74.12) |
| naringenin-7-O-glucoside | 5.50 | 434.1213 | 433.1158 | 434.1228 | 3.46 | AM & Rt |
| prunin 6''-p-coumarate | 2.97 | 580.1581 | 579.1509 | 580.1583 | 0.18 | AM & ID(99.37) |
|  |  |  |  |  |  |  |
| *Flavonols* |  |  |  |  |  |  |
| kaempferol | 7.72 | 286.0477 | 285.0406 | 286.0478 | 0.35 | AM & Rt |
| myricetin | 6.37 | 318.0376 | 317.0303 | 318.0377 | 0.04 | AM & Rt |
| dihydromyricetin | 3.70 | 320.0532 | 319.0446 | 320.0519 | 4.01 | AM & ID(96.52) |
| p-coumaroyl quinic acid | 3.43 | 338.1002 | 337.0932 | 338.1003 | 0.14 | AM & ID(96.64) |
| kaempferol-3-O-glucoside | 5.88 | 448.1006 | 447.0926 | 448.1005 | 0.22 | AM & Rt |
| quercetin-3-O-rhamnoside | 5.48 | 448.1006 | 447.0953 | 448.1028 | 4.91 | AM & Rt |
| kaempferol 3-β-d-glucopyranoside | 5.53 | 448.1006 | 447.0963 | 448.1036 | 6.69 | AM & Rt |
| quercetin-3-O-galactoside | 4.70 | 464.0955 | 463.0887 | 464.0959 | 0.86 | AM & Rt |
| quercetin-3-O-glucoside | 4.75 | 464.0955 | 463.0875 | 464.0948 | 1.42 | AM & Rt |
| isoquercitrin | 4.85 | 464.0955 | 463.0875 | 464.0948 | 1.51 | AM & Rt |
| quercetin 3-(3''-acetylrhamnoside) | 4.77 | 490.1111 | 489.1032 | 490.1105 | 1.31 | AM & ID(97.69) |
| kaempferol -(2''-p-coumaryl-alpha-L-arabinopyranoside) | 7.39 | 564.1268 | 563.1199 | 564.1272 | 0.71 | AM & ID(98.01) |
| quercetin -(2''-galloyl-alpha-L-arabinopyranoside) | 4.25 | 586.0959 | 585.0869 | 586.0943 | 1.56 | AM & ID(93.76) |
| kaempferol-3-O-rutinoside | 5.50 | 594.1585 | 593.1526 | 594.1593 | 1.35 | AM & Rt |
| quercetin-3-Glc-Ara | 4.23 | 596.1377 | 595.1311 | 596.1387 | 1.68 | AM & Rt |
| quercetin 3-xyloside-7-glucoside | 3.49 | 596.1377 | 595.1311 | 596.1388 | 1.82 | AM & ID(96.04) |
| kaempferol 7-(6''-galloylglucoside) | 4.82 | 600.1115 | 599.1059 | 600.1130 | 1.55 | AM & ID(94.84) |
| rutin | 4.50 | 610.1534 | 609.1470 | 610.1538 | 0.66 | MS/MS |
| myricetin 3-sambubioside | 2.99 | 612.1326 | 611.1251 | 612.1324 | 0.32 | AM & ID(92.38) |
| myricetin 3-(3''-galloylrhamnoside) | 4.25 | 616.1064 | 615.0985 | 616.1060 | 0.71 | AM & ID(96.57) |
| quercetin 3,4'-diglucoside | 3.90 | 626.1483 | 625.1417 | 626.1490 | 1.12 | AM & Rt |
| robinin | 3.98 | 740.2164 | 739.2125 | 740.2196 | 4.32 | AM & Rt |
| quercetin 3-(3R-glucosylrutinoside) | 4.12 | 772.2062 | 771.2003 | 772.2074 | 1.24 | AM & ID(97.41) |
| kaempferol 3-(4''-caffeyllaminaribioside)-7-rhamnoside | 7.13 | 918.2430 | 917.2363 | 918.2435 | 0.56 | AM & ID(96.52) |
|  |  |  |  |  |  |  |
| *Flavones* |  |  |  |  |  |  |
| 7-methoxyflavone | 3.83 | 252.0786 | 291.0430 | 252.0801 | 6.07 | AM & ID(88.5) |
| luteolin | 8.69 | 286.0477 | 285.0404 | 286.0481 | 1.40 | AM & Rt |
| morin | 6.56 | 302.0427 | 301.0354 | 302.0426 | 0.05 | AM & Rt |
| 3-hydroxy-7,8,4'-trimethoxyflavone | 4.31 | 328.0947 | 327.0876 | 328.0947 | 0.06 | AM & ID(96.74) |
| linderoflavone A | 2.99 | 358.0689 | 357.0621 | 358.0695 | 1.63 | AM & ID(95.57) |
| luteolin-7-O-glucoside | 4.96 | 448.1006 | 447.0952 | 448.1022 | 3.57 | AM & Rt |
| luteolin-8-C-glucoside | 4.00 | 448.1006 | 447.0912 | 448.0979 | 6.03 | AM & Rt |
|  |  |  |  |  |  |  |
| *Flavan-3-ols* |  |  |  |  |  |  |
| catechin | 3.00 | 290.0790 | 291.0861/289.0719 | 290.0791 | 0.44 | Standard |
| epicatechin | 3.63 | 290.0790 | 291.0862/289.0718 | 290.0790 | 0.02 | Standard |
| pelargonidin | 2.39 | 306.0295 | 307.0381 | 306.0306 | 3.71 | AM & ID(65.24) |
| epigallocatechin | 2.72 | 306.0740 | 307.0814/305.0671/611.1407 | 306.0741 | 0.46 | Standard |
| gallocatechin | 2.04 | 306.0740 | 305.0651 | 306.0726 | 4.57 | Standard |
| qurateacatechin | 5.08 | 320.0896 | 319.0825 | 320.0898 | 0.65 | AM & ID(99.09) |
| leucodelphinidin | 2.34 | 322.0689 | 321.0601 | 322.0674 | 1.47 | AM & ID(92.96) |
| epicatechin 5,7,3'-trimethyl ether | 7.42 | 332.1260 | 331.1176 | 332.1251 | 2.82 | AM & ID(91.52) |
| catechin 7-O-apiofuranoside | 5.17 | 422.1213 | 421.1141 | 422.1213 | 0.03 | AM & ID(99.75) |
| epigallocatechin 3-O-(4-hydroxybenzoate) | 5.89 | 426.0951 | 425.0874 | 426.0967 | 1.56 | AM & ID(93.63) |
| catechin 7-O-alpha-L-rhamnopyranoside | 5.48 | 436.1369 | 435.1297 | 436.1370 | 0.33 | AM & ID(98.68) |
| epicatechin gallate | 3.70 | 442.0900 | 443.0968/465.0754/441.0903/883.1719 | 442.0895 | 1.13 | Standard |
| catechin gallate | 4.70 | 442.0900 | 441.0832/883.1728/443.0973/465.0789 | 442.0900 | 0.63 | Standard |
| epicatechin 8-C-glucoside | 8.32 | 452.1319 | 451.1237 | 452.1308 | 2.51 | AM & ID(94.12) |
| epicatechin 3-O-(3-O-methylgallate) | 5.82 | 456.1056 | 455.0981 | 456.1043 | 1.32 | AM & ID(85.12) |
| gallocatechin gallate | 3.72 | 458.0849 | 457.0773/459.0918 | 458.0845 | 0.87 | Standard |
| epigallocatechin gallate | 3.67 | 458.0849 | 459.0923/457.0777/915.1624 | 458.0852 | 0.58 | Standard |
| epigallocatechin 3-O-caffeate | 5.14 | 468.1056 | 467.0983 | 468.1057 | 0.31 | AM & ID(98.31) |
| catechin-4-ol 3-O-beta-D-galactopyranoside | 2.45 | 468.1268 | 467.1193 | 468.1264 | 0.94 | AM & ID(98.03) |
| epigallocatechin 3-(4-methyl-gallate) | 4.24 | 472.1006 | 471.0955 | 472.1035 | 2.89 | AM & ID(91.38) |
| epicatechin pentaacetate | 6.91 | 500.1319 | 499.1251 | 500.1322 | 0.61 | AM & ID(95.34) |
| procyanidin A2 | 3.20 | 578.1424 | 577.1383 | 578.1456 | 5.53 | MS/MS |
| procyanidin B1 | 2.62 | 578.1424 | 577.1365 | 578.1438 | 2.42 | AM & Rt |
| procyanidin B2 | 2.64 | 578.1424 | 577.1369 | 578.1446 | 3.81 | AM & Rt |
| procyanidin B3 | 2.79 | 578.1424 | 577.1377 | 578.1446 | 3.81 | AM & Rt |
| catechin 5,7,-di-O-gallate | 6.00 | 594.1010 | 593.0943 | 594.1015 | 0.55 | AM & ID(98.63) |
| catechin 3-O-rutinoside | 4.74 | 598.1898 | 597.1828 | 598.1901 | 0.32 | AM & ID(99.35) |
| cyanidin 3-(6''-caffeyl glucoside) | 2.15 | 610.1323 | 609.1233 | 610.1308 | 1.48 | AM & ID(88.04) |
| cyanidin-3-O-(6''-O-malonyl-2''-O-glucuronyl) glucoside | 1.45 | 710.1330 | 709.1264 | 710.1336 | 0.85 | AM & ID(96.53) |
| ent-epicatechin-(4 alpha->8)-ent-epicatechin 3'-gallate | 3.83 | 730.1534 | 729.1458 | 730.1529 | 0.46 | AM & ID(99.61) |
| epicatechin-(4beta->8)-epigallocatechin 3-O-gallate | 3.32 | 746.1483 | 745.1428 | 746.1493 | 0.97 | AM & ID(94.14) |
| epigallocatechin gallate 7-glucoside | 4.11 | 796.1698 | 795.1637 | 796.1711 | 1.60 | AM & ID(88.82) |
| epiafzelechin-(4beta->6)-epicatechin 3,3'-digallate | 6.98 | 866.1694 | 865.1634 | 866.1702 | 0.82 | AM & ID(97.74) |
| cinnamtannin A1 | 3.66 | 866.2058 | 865.1992 | 866.2060 | 0.27 | AM & ID(98.29) |
|  |  |  |  |  |  |  |
| *Isoflavonoids* |  |  |  |  |  |  |
| dipteryxine | 0.84 | 342.0740 | 365.0626 | 342.0733 | 2.19 | AM & ID(80.76) |
|  |  |  |  |  |  |  |
| *Terpenoid alkaloids* |  |  |  |  |  |  |
| caffeine | 3.31 | 194.0804 | 195.0908 | 194.0829 | 12.88 | MS/MS |
| elatine | 3.46 | 694.3465 | 717.3361 | 694.3416 | 7.07 | AM & ID(80.63) |

^a^ Peaks were identified on the basis of actual mass (AM) and MS/MS, MS/MS data matched with online metabolite databases;

^b^ Peaks were identified on the basis of AM and retention time (RT), the retention time of polyphenol compared with the published literature (Vrhovsek et al., 2012);

^c^ Peaks were identified on the basis of AM and isotopic distribution (ID), the number shows the score of matched with MassHunter;

^d^ Peaks were identified on the basis of AM, RT and Standard;

The identified peaks may be further classified into Identified compounds (b and d) and Putatively annotated compounds (a and c) according to the proposed minimum reporting standards for chemical analysis (Sumner,L.W. et al., 2007).
