# Supplementary material for: Metabolomic Analysis Using Ultra-Performance Liquid Chromatography-Quadrupole-Time of Flight Mass Spectrometry (UPLC-Q-TOF MS) Uncovers the Effects of Light Intensity and Temperature under Shading Treatments on the Metabolites in Tea
Source: PLoS One. 2014 Nov 12;9(11):e112572. doi: 10.1371/journal.pone.0112572 (PMC4229221; doi:10.1371/journal.pone.0112572)
Supplement: Table S3 — The unidentified key components differentiating the two shading treatments (Black Net, B; Nano-insulating Film, N) and the un-shaded control (CK). (DOC) [file pone.0112572.s003.doc]

Table S3 The unidentified key components differentiating the two shading treatments (Black Net, B; Nano-insulating Film, N) and the un-shaded control (CK).

| Peak | RT(min) | Mol.ion m/z(±) | N/CK | | B/CK | | N/B | | MS/MS^c^ |
| --- | --- | --- | --- | --- | --- | --- | --- | --- | --- |
|  |  |  | VIP^a^ | Fold^b^ | VIP | Fold | VIP | Fold |  |
| 1 | 0.74 | 383.1133[M-H]^-^ | 3.88 | 0.34 | 3.78 | 0.49 | 2.92 | 0.70 | 191.00 |
| 2 | 0.74 | 533.1709[M-H]^-^ | 2.65 | 0.44 | 2.60 | 0.52 |  |  | 191.00 |
| 3 | 0.75 | 192.0589[M-H] | 3.40 | 0.45 | 3.18 | 0.60 | 2.89 | 0.74 |  |
| 4 | 0.76 | 104.1062[M-H] |  |  |  |  | 2.86 | 1.07 |  |
| 5 | 0.81 | 114.9999[M-H]^-^ |  |  |  |  | 2.09 | 1.17 |  |
| 6 | 0.92 | 130.0429[M+H]^+^ | 2.22 | 1.37 | 2.89 | 1.50 |  |  | 114.00 |
| 7 | 0.92 | 176.0935[M-H]^-^ | 2.10 | 1.75 | 2.47 | 1.84 |  |  |  |
| 8 | 0.98 | 337.1457[M-H]^-^ | 3.91 | 0.76 |  |  | 6.72 | 0.77 | 319, 301, 253, 158 |
| 9 | 0.98 | 290.0882[M-H]^-^ |  |  |  |  | 2.76 | 0.73 |  |
| 10 | 0.99 | 335.1436[M-H]^-^ |  |  | 3.04 | 1.42 | 5.51 | 0.59 | 173, 155 |
| 11 | 1.00 | 671.2971[M-H]^-^ |  |  |  |  | 2.32 | 0.43 |  |
| 12 | 1.00 | 245.1119[M-H]^-^ |  |  |  |  | 2.67 | 0.64 |  |
| 13 | 1.00 | 128.0374[M-H]^-^ | 2.45 | 1.60 | 3.08 | 1.75 |  |  | 100.00 |
| 14 | 1.01 | 100.0757[M-H]^-^ |  |  | 2.06 | 2.40 | 2.08 | 0.73 |  |
| 15 | 1.20 | 331.0682[M-H]^-^ | 2.50 | 1.18 | 2.89 | 1.21 |  |  | 169, 211, 271 |
| 16 | 1.22 | 229.1326[M-H]^-^ |  |  |  |  | 2.58 | 1.15 |  |
| 17 | 1.35 | 201.0869[M-H]^-^ |  |  |  |  | 2.09 | 0.72 |  |
| 18 | 1.41 | 289.1037[M-H]^-^ | 2.85 | 3.14 | 2.65 | 2.74 |  |  | 271, 253, 173, 155 |
| 19 | 1.51 | 169.0162[M-H]^-^ |  |  | 2.87 | 1.18 | 2.53 | 0.94 | 125.00 |
| 20 | 1.58 | 130.0781[M+H]^+^ | 4.85 | 3.47 | 5.04 | 3.17 | 2.34 | 1.10 | 103.00 |
| 21 | 1.67 | 245.1231[M+H]^+^ |  |  |  |  | 2.00 | 0.72 | 200, 158.0804, 126 |
| 22 | 1.76 | 395.0309[M-H]^-^ | 3.09 | 2.11 | 3.41 | 2.09 |  |  | 351, 241, 153, 109 |
| 23 | 1.80 | 120.0759[M+H]^+^ | 2.83 | 0.61 | 2.26 | 0.80 | 2.99 | 0.77 | 103.00 |
| 24 | 2.41 | 458.0675[M+H]^+^ | 2.48 | 4.85 | 2.52 | 4.21 |  |  | 320, 239, 288.0867, 139 |
| 25 | 2.98 | 634.0808[M-H]^-^ | 2.12 | 1.68 |  |  | 3.04 | 1.42 | 300.00 |
| 26 | 3.13 | 124.0207[M-H]^-^ |  |  |  |  | 2.44 | 1.92 |  |
| 27 | 3.15 | 349.0713[M-H]^-^ | 3.02 | 2.03 | 2.66 | 1.66 | 2.93 | 1.23 |  |
| 28 | 3.15 | 616.1326[M-H]^-^ |  |  | 2.35 | 2.44 |  |  |  |
| 29 | 3.17 | 309.1058[M-H]^-^ |  |  | 2.16 | 2.85 | 2.12 | 0.65 |  |
| 30 | 3.19 | 196.0815[M-H]^-^ | 2.99 | 1.24 | 2.98 | 1.20 |  |  | 138, 111 |
| 31 | 3.31 | 715.1926[M-H]^-^ |  |  |  |  | 2.16 | 0.67 |  |
| 32 | 3.55 | 611.1205[M-H]^-^ | 2.39 | 1.49 | 2.57 | 1.46 |  |  | 329, 491, 449, 397, 517 |
| 33 | 3.67 | 795.1802[M-H]^-^ |  |  | 2.36 | 0.89 |  |  |  |
| 34 | 3.69 | 744.1736[M-H]^-^ |  |  |  |  | 2.27 | 0.57 |  |
| 35 | 3.82 | 418.1120[M+H]^+^ |  |  |  |  | 3.78 | 0.50 | 250, 139, 327 |
| 36 | 3.95 | 565.1097[M+H]^+^ | 2.32 | 0.61 |  |  | 2.59 | 0.74 | 427, 511, 379, 457, 325 |
| 37 | 4.06 | 481.0587[M-H]^-^ | 2.68 | 0.58 | 2.73 | 0.64 |  |  | 319.00 |
| 38 | 4.25 | 439.1723[M-H]^-^ |  |  |  |  | 2.01 | 1.42 |  |
| 39 | 4.25 | 593.1529[M-H]^-^ |  |  |  |  | 2.85 | 0.84 | 479, 445, 541, 501 |
| 40 | 4.37 | 487.0975[M-H]^-^ |  |  |  |  | 2.08 | 2.26 |  |
| 41 | 4.48 | 579.1139[M-H]^-^ | 3.09 | 0.60 | 2.65 | 0.74 | 2.81 | 0.81 |  |
| 42 | 4.56 | 568.1478[M-H]^-^ |  |  | 2.94 | 2.59 | 3.25 | 0.64 | 169, 236, 125, 416 |
| 43 | 4.63 | 570.1088[M-H]^-^ |  |  | 2.43 | 1.86 | 3.08 | 0.66 |  |
| 44 | 4.80 | 261.1598[M-H]^-^ | 2.79 | 1.29 |  |  | 3.67 | 1.17 | 244, 202, 131 |
| 45 | 5.89 | 552.1543[M-H]^-^ |  |  | 3.00 | 1.95 | 3.66 | 0.65 | 400, 169 |
| 46 | 5.98 | 851.1843[M-H]^-^ |  | 2.07 |  | 1.28 | 2.02 | 1.62 | 425, 788, 169, 684 |
| 47 | 6.83 | 1079.2861[M-H]^-^ | 3.17 | 0.23 | 3.28 | 0.33 |  |  | 301, 933, 758, 451, 178 |
| 48 | 6.93 | 343.2119[M-H]^-^ | 2.88 | 1.66 | 2.78 | 1.53 |  |  | 229, 209, 171, 113 |
| 49 | 7.94 | 493.2302[M-H]^-^ | 2.20 | 1.63 |  |  | 2.89 | 1.35 | 447, 315, 101, 341, 169 |
| 50 | 8.90 | 327.2166[M-H]^-^ | 3.57 | 1.85 | 3.34 | 1.66 | 2.06 | 1.11 | 291, 229, 211, 183, 171 |
| 51 | 9.35 | 329.2340[M-H]^-^ | 4.86 | 1.98 | 4.34 | 1.71 | 3.38 | 1.16 | 211, 171, 139, 127 |
| 52 | 10.35 | 307.1869[M-H]^-^ | 2.84 | 2.40 |  |  | 3.12 | 1.44 | 291, 251, 171, 109 |
| 53 | 10.36 | 291.1665[M+H]^+^ | 2.05 | 1.89 |  |  | 2.10 | 1.30 | 135, 163 217 |
| 54 | 10.70 | 449.2301[M-H]^-^ | 2.28 | 2.13 |  |  | 2.76 | 1.47 | 152, 295 |
| 55 | 10.95 | 721.3685[M-H]^-^ |  |  |  |  | 2.85 | 1.50 | 675, 397, 277, 235 |
| 56 | 11.07 | 593.2849[M-H]^-^ |  |  |  |  | 2.30 | 1.62 | 277, 152, 315, 241 |
| 57 | 11.08 | 364.2785[M+H]^+^ | 2.19 | 3.72 |  |  | 2.46 | 1.71 | 305.00 |
| 58 | 11.26 | 293.2103[M-H]^-^ | 3.66 | 1.86 |  |  | 4.69 | 1.51 | 275, 183, 195 |
| 59 | 11.34 | 342.2995[M+H]^+^ | 2.36 | 3.24 | 2.14 | 2.64 |  |  | 283.00 |
| 60 | 11.35 | 277.1882[M+H]^+^ | 2.56 | 1.88 |  |  | 3.04 | 1.43 | 107, 121, 135, 147, 157 |
| 61 | 11.35 | 577.2753[M-H]^-^ | 2.66 | 2.00 | 2.48 | 1.17 | 3.84 | 1.70 | 255, 152, 305, 446 |
| 62 | 11.42 | 496.2904[M+H]^+^ |  |  |  |  | 2.21 | 0.75 | 184, 313, 104 |
| 63 | 11.48 | 295.2178[M-H]^-^ | 3.89 | 2.15 | 2.21 | 1.45 | 4.57 | 1.49 | 277, 195, 171 |
| 64 | 11.54 | 265.1488[M-H]^-^ |  |  |  |  | 2.94 | 0.61 |  |
| 65 | 11.56 | 431.2243[M-H]^-^ |  |  |  |  | 2.75 | 2.13 | 152, 277 |
| 66 | 12.92 | 607.2453[M+H]^+^ |  |  |  |  | 2.32 | 0.73 | 461.00 |
| 67 | 14.01 | 409.2369[M-H]^-^ | 2.34 | 1.82 |  |  | 2.87 | 1.39 |  |
| 68 | 14.02 | 433.2340[M-H]^-^ |  |  |  |  | 2.78 | 1.76 |  |

^a^ The fold change value is based on comparing the peak intensity (content of metabolites) between different treatments (groups).

^b^ VIP is Variable Importance in the Projection. Key components were obtained by filtering with the VIP >1 and P < 0.001 in the statistical analysis.

^c^ MS/MS fragmentation process was accomplished at a normalized collision energy of 10 eV by UPLC-Q-TOF MS to create information about molecular weight.
